# Psychometric properties of the Malay version of the Illness Cognition Questionnaire among cancer patients in Malaysia

**DOI:** 10.1186/s12889-023-17060-1

**Published:** 2024-01-13

**Authors:** Wenjun Song, Nor Shuhada Mansor, Nurul Izzah Shari, Ruiling Zhang, Mohammad Farris Iman Leong Bin Abdullah

**Affiliations:** 1 The Second Affiliated Hospital of Xinxiang Medical University, Henan, People’s Republic of China; 2 Department of Community Health, Advanced Medical and Dental Institute, Universiti Sains Malaysia, Kepala Batas, Pulau Pinang, 13200 Malaysia; 3 School of Human Resource Development and Psychology, Faculty of Social Sciences and Humanities (FSSH), Universiti Teknologi Malaysia, Skudai, Johor, 81310 Malaysia

**Keywords:** Malay version illness cognition questionnaire, Reliability, Validity, Cancer patients

## Abstract

**Objective:**

The Illness Cognition Questionnaire (ICQ) was translated from its original English version to the Malay version for this research, adapted the Malay language version of the ICQ (ICQ-M) for use in cancer patients, and assessed the internal consistency, content, face, construct, convergent, discriminant and concurrent validity of the ICQ-M among a cohort of cancer patients with mixed cancer types in Malaysia.

**Method:**

Initially, the ICQ was translated into Malay and back-translated, and its content and face validity were evaluated. Then, 346 cancer patients with various cancer types received the ICQ-M, and its internal consistency, convergent, discriminant, construct, and concurrent validity were evaluated.

**Results:**

The ICQ-M and its domains had acceptable internal consistency with Cronbach’s α ranging from 0.742 to 0.927. Construct validity assessment demonstrated that the ICQ-M consists of 17 items designated in two domains with good convergent and discriminant validity. The ICQ-M and its domains also had moderate correlations with the Acceptance and Action Questionnaire II, which denotes that the ICQ-M had acceptable concurrent validity.

**Conclusion:**

The ICQ-M had good psychometric properties and is now available to measure the illness cognition of cancer patients in Malaysia.

## Introduction

Disease cognition, or disease perception, refers to the arousal of one’s psychological coping response through the individual’s cognitive evaluation and emotional expression of the disease when the disease state leads to threatened health status [1]. Studies have found that patients’ cognition of the disease will affect their coping and adjustment, such as health behaviour toward treatment, treatment compliance, and emotion [2, 3]. This negative effect may directly or indirectly influence the prognosis of the illness, the patient's quality of life, and their capacity for social interaction [4, 5].

Cancer patients, irrespective of the specific cancer type, often experience various emotions stemming from their diagnosis and treatment journey, including fear, anxiety, and uncertainty [6, 7]. The depth of their knowledge and cognitive understanding of cancer significantly shapes their coping strategies and emotional responses. With the advancement of cancer diagnosis and treatment, the survival rate of cancer patients has increased significantly, and the survival period has been prolonged [8]. In cancer rehabilitation, understanding and measuring disease cognition play a pivotal role [9]. Therefore, investigating and measuring disease cognition in cancer patients is crucial.

Two commonly used tools for assessing patients' cognition perception and cognition of their illness are the Brief Illness Perception Questionnaire (B-IPQ) and the Illness Cognition Questionnaire (ICQ). IPQ is based on Weinman et al. (1996) self-regulatory model (SRM) theory [10]. SRM divides patients' cognition of disease into five aspects: disease identity, disease continuity, disease control, pathogenic factors, and serious consequences [10]. IPQ has been widely used in patients with various chronic diseases and has good reliability and validity in breast cancer patients [11]. While the Illness Perception Questionnaire-Revised (IPQ-R) consists of 80 items which is not suitable to assess cancer patients who are symptomatic and in physical distress as well as those who are short of time. B-IPQ is a much shorter version of the original IPQ which consists of 9 items rated from Likert scale of 0 (minimum) to 10 (maximum) [12]. ICQ was compiled by Evers et al. (2001). It is used to assess patients' cognition of the stress and disgust characteristics of the disease at the psychological and behavioural level within three dimensions: helplessness, acceptance, and perceived benefits [13]. The questionnaire has good reliability and validity among patients with chronic diseases, such as chronic pain, fatigue, and rheumatoid arthritis [8, 13]. The differences between B-IPQ and ICQ include: (1) B-IPQ has 9 items, while ICQ has 18 items; (2) the B-IPQ items assess cognitive perceptions of illness, emotional aspect of illness, degree of understanding of illness and causes of illness; while the ICQ assess acceptance, perceived benefits and helplessness concerning the illness experienced; and (3) the B-IPQ is intended for use in groups and hence it is more suitable for use in research, whereas the ICQ is good for use in groups as well as in individuals and therefore suitable for use in research and clinical setting [12, 13]. Aa a result of wider application of the ICQ, it is more crucial to translate and validate the ICQ compared with B-IPQ. At present, there is a lack of data regarding the reliability and validity of the ICQ when used with cancer patients in Malaysia.

Nevertheless, conducting cultural adaptation and validation studies is crucial to ensure the ICQ's relevance and accuracy across diverse cultural contexts. Given Malaysia's distinct sociocultural background and rising cancer rates, it presents a compelling context to validate the ICQ among cancer patients. Validating a Malay version of the ICQ is highly significant as it offers a culturally relevant and linguistically valid tool to evaluate perceived social support among Malay-speaking cancer patients in Malaysia. Therefore, it is essential to translate the ICQ into the Malay version. Firstly, translating the ICQ into Malay improves accessibility and promotes a better understanding of illness cognition among individuals who primarily speak Malay. Secondly, cultural relevance is essential to consider. Translating the questionnaire into Malay ensures that the questions and scales are culturally appropriate for individuals in Malaysia who may hold different beliefs, attitudes, and practices related to health and illness. Furthermore, a Malay version of the ICQ enables clinicians and health professionals to effectively address cognitive distortions associated with illness in Malay-speaking patients, who form a significant population in Malaysia. Additionally, the availability of a Malay version of the ICQ facilitates research on illness cognition within the Malay-speaking population, contributing to a deeper understanding of cultural factors influencing illness beliefs and coping strategies in Malaysia. Translating the ICQ into Malay enhances research outcomes on illness cognition, thereby improving the quality of healthcare for cancer patients in Malaysia. As a result, this validation study translated the original English language version of the ICQ into the Malay language version, modified it for use in cancer patients, and evaluated the reliability and validity of the Malay version of the ICQ (ICQ-M) in a cohort of cancer patients with mixed diagnoses in Malaysia to fill the research gap.

## Materials and methods

### Study design and study sample

The validation study took place from December 2022 to March 2023. The study focused on cancer patients registered under the oncology unit of the Advanced Medical and Dental Institute (AMDI). It is a tertiary medical facility for oncology in Peninsular Malaysia, covering states like Pulau Pinang, Kedah, Perlis, and Perak. The AMDI, USM oncology unit currently has approximately 900 to 1000 registered oncology patients with various cancer diagnoses.

The required sample size for this validation research was established using a sample size calculator. Using an online sample size calculator (https://wnarifin.github.io/ssc/ssalpha.html), the estimated sample size needed for meaurement of internal consistency by Cronbach’s α was carried out: the minimum acceptable Cronbach's alpha was 0.7, the estimated Cronbach's alpha was 0.8 [8], the type I error was 0.05, the power was 0.8, and the number of questionnaire items was 18. Therefore, 130 participants (with 20% dropout) were deemed the minimum required to evaluate internal consistency. Finally, the estimated sample size required for confirmatory factor analysis was calculated using an A priori sample size calculator for the structural equation model (https://www.danielsoper.com/statcalc/calculator.aspx?id=89), whereby the type I error of 0.05, a power of 0.8, three latent variables, eighteen observable variables, and the effect size was 0.22 [14]. The estimated sample size required for the study was determined to be 318 subjects, taking into account a 20% anticipated dropout rate. This sample size was determined based on the requirement to assess confirmatory factor analysis, which yielded the largest estimated sample size among all the calculations. Thus, the final sample size required for the study was established at 318 subjects. The sample size required for CFA assessment of instrument may depends on the number of indicators per factor. A CFA model with 6—12 indicator variables per factor would required a sample size of at least 50 subjects. A CFA model with 3 to 4 indicators per factor would need a sample size of at least 100 subjects. While a CFA model with only 2 indicators per factor would required more than 400 subjects [15]. The original ICQ has 6 indicators per factor and hence, a sample size of 318 subjects is deemed as sufficient.

The subject recruitment for the study was carried out using consecutive sampling [16]. The research team explained the investigation by interviewing cancer patients who attended the outpatient clinic and in-patient ward of the AMDI, USM oncology unit. Then, potential subjects were screened with the eligibility criteria of the study. The inclusion criteria were: (1) patients with any cancer with a diagnosis confirmed by the histopathological report and at any stage and any duration since diagnosis, (2) those with age from 18 years old and above, (3) those who could read and write in Malay, (4) those who were cognitively sound to answer questionnaires and (5) those who are physically capable of answering the questionnaire. Participants in the research must fulfill all inclusion criteria to be invited.

### Translation and back translation of the ICQ-M and content validity

Two independent language specialists who are multilingual native Malay speakers from the School of Language and Literacy at one institution translated the ICQ's original English edition into Malay. Subsequently, a bilingual language expert from the same institution, fluent in English without seeing the original version of the ICQ, performed a back-translation from the Malay language into English. To establish the content validity of the translated Malay version, a team of experts made up of a psychiatrist, two psychologists, and two community health professionals evaluated the translation and back-translation draughts of the ICQ (the two community health professionals were included in the panel of experts as their area of research also focus on the mental health aspect of cancer patients in the community). The selection criteria for the panel of experts were: (1) engaged in mental health research in cancer patients for at least 5 years after completion of postgraduate qualification, (2) academic qualification with Master of Medicine or PhD, and (3) voluntary participation in the study. The panel's experts were individually asked to assess the relevance of each item to the ICQ's domains using the available answer choices. The response options are: (1) the item is not relevant to the measured domain, (2) the item is partially relevant to the measured domain, (3) the item is relevant to the measured domain, and (4) the item is very relevant to the measured domain. Those who rated an item with response option (3) and (4) was given a score of 1, while those who placed an item with response option (1) and (2) was assigned a score of 0. By dividing the total number of experts in the panel by the number of assessors who received a score of 1, it will produce the item-level content validity index (I-CVI). An I-CVI value of > 0.83 was acceptable [17, 18]. An item’s universal agreement (UA) is equal to 1 if all the experts in the panel agree that the item is either “relevant to the domain measured” or “very relevant to the domain measured”; otherwise, its UA is scored 0. The scale level content validity index (S-CVI/UA) is calculated as the total sum of UA divided by the complete items of the ICQ, in which a value of > 0.8 is considered as high [19]. At the same time, I-CVI is added together and divided by the total number of ICQ items to get the average scale-level content validity index(S-CVI/Ave), whereby > 0.9 is deemed to be high [20]. After the panel of experts examined the drafted translations and back-translated copies of the ICQ, the drafted Malay language version of the ICQ (ICQ-M) was constructed.

The draft of the ICQ-M was then given to twenty Malay-speaking cancer patients to evaluate the face validity. All the twenty subjects were interviewed individually for their assessment of the semantic quality, the comprehensibility of the words and sentences used in the instructions and items, any repetition or redundance of terms and sentences, and the duration of the administration. Their responses to each of the four factors mentioned above were coded as “inappropriate,” “appropriate,” and “very appropriate” after the interview.

### Measures

The participants enrolled in the study were administered the socio-demographic and clinical characteristics questionnaire, the ICQ-M, and the Malay language version of the acceptance and action questionnaire II (AAQ-II).

#### Socio-demographic and clinical characteristics questionnaire

The socio-demographic and clinical characteristics questionnaire gathered information on various variables, such as gender, age, religion, monthly family income, ethnicity, marital status, level of education, types of cancer, duration since diagnosis, and cancer stage.

The response options to the age of the participants were coded as “18–25 years”, “26–45 years”, “46–65 years”, and “more than 65 years”. The response options to gender were coded as “male” and “female.” The response options to ethnicity were coded as “Malay,” “Chinese,” “Indian,” and “others.” The response options to religion were coded as “Islam,” “Buddhism,” “Hindu,” and “Christian.” The response options to monthly household income were coded into “less than RM 4500”, “between RM 4500 to RM 11000”, and “more than RM 11000”. The response options to the participants’ marital status were coded as “married” as well as “single/divorced/widow/widower.” For the education status options, the answer possibilities were "tertiary education and above," " up to secondary education," and "primary education and below."

Regarding the clinical characteristics, the types of cancer were coded as "breast cancer," "lung cancer," "head and neck cancer," "colorectal cancer," and "others." The time since diagnosis options were coded as "less than three months," "3–6 months," "6–12 months," "1–2 years," and "more than two years." The stage of cancer options included "stage 1," "stage 2," "stage 3," and "stage 4."

#### Illness Cognitive Questionnaire (ICQ)

The ICQ, which is self-administered tool which is used to evaluate how people with different chronic conditions perceive themselves [21]. It comprises of 18 items allocated to three domains: acceptance, perceived benefits, and helplessness. Each domain consists of 6 items, each scored on a Likert scale of 1 = not at all to 4 = completely. Hence, the domain score varies between 6 and 24. The degree of the assessed domain increases with increasing domain score. The domains of the ICQ have good to excellent internal consistency with Cronbach’s α between 0.84 and 0.91.

#### Acceptance and Action Questionnaire II (AAQ-II)

The AAQ-II is a self-administered tool to assess experiential avoidance or psychological inflexibility. Psychological inflexibility is the lack of ability to accept and adapt to difficult life situations by fully experiencing the present moment and consciously selecting value-consistent behaviour in response, regardless of the person's internal experience. The AAQ II is the second version, improved from the first edition. It is shorter (7 items) and has good psychometric properties. The seven items were added together to determine the scores. With higher scores came more psychological rigidity [22]. The Malay version of the AAQ-II [AAQ-II (M)] was validated, and results showed that it was a unidimensional scale that examined psychological inflexibility/experiential avoidance among cancer patients in Malaysia. Cronbach's alpha was 0.91, and the instrument had great internal consistency [23]. This research evaluated the concurrent validity of the ICQ-M using the AAQ-II (M) as a gold standard comparator.

### Ethical consideration

This study was approved by the Human Research Ethics Committee of Universiti Sains Malaysia (ethics code: USM/JEPeM/22080569), adhering to the Helsinki Declaration of 1964 regulations and its subsequent amendments. A comprehensive explanation of the study's objectives, procedures, potential benefits and risks, participants' right to withdraw at any time, and the assurance of data anonymity was provided. Participants signed informed consent to join the study after receiving this information, with the understanding that their collected data would be discarded after completion of the study.

### Statistical analysis

We used SPSS version 26 (SPSS Inc., Chicago, IL, USA) for data analysis, except confirmatory factor analysis (CFA), which was performed using SPSS Amos version 26 software. Descriptive statistics were reported for sociodemographic and clinical characteristics, while mean scores were calculated for the ICQ domains. Continuous data were reported as mean, standard deviation (SD), skewness and kurtosis; whereas categorical variables were provided as frequency and percentage. The continous data (total and domain scores of ICQ-M) were normally distributed. There was no missing data in this study.

Construct validity was evaluated using confirmatory factor analysis (CFA). The method employed to estimate parameters in CFA was maximum likelihod method. In this method, the measurement model for CFA is a multivariate regression model which describe the relationship between a set of observed dependent variables and a set of continous latent variables. Here, the observed dependent variables were factor indicators, while the continous latent variables were factors. Several parameters were compared across several ICQ-M models to find the model that best suited the ICQ-M (such as: (a) 3-factor model with item allocation and factor structure similar to the original English version of the ICQ; (b) 2-factor model with merging of the acceptance and perceived benefit domains into a single domain, while the helplessness domain as another domain of the ICQ-M and all 18 items included; and (c) with merging of the acceptance and perceived benefit domains into a single domain, while the helplessness domain as another domain of the ICQ-M and item 7 omitted). The chi-square to degrees of freedom ratio ( ^2^/df) of 2.0 with a *p*-value > 0.05, the Tucker-Lewis index (TLI) of 0.95, the comparative fit index (CFI) of 0.95, the goodness of fit index (GFI) of 0.90, and the root mean square error of approximation (RMSEA) of 0.06 were the parameters used to evaluate the model fit [20, 24]. During the CFA evaluation, the above criteria were considered acceptable in determining the most suitable factor model for the ICQ-M.

The convergent and discriminant validity of the ICQ-M were evaluated using the confirmatory factor analysis (CFA) best-fitting model. Convergent validity was assessed by calculating the average variance extracted (AVE), which required adding up the squared factor loadings of items within a certain domain and dividing it by the total number of indicators. An AVE value greater than 0.5 indicated that the ICQ-M demonstrated convergent validity [20, 25]. The square root of the AVE for a specific domain and the inter-construct correlation coefficients across domains were compared to evaluate the model's discriminant validity. The ICQ-M had gained discriminant validity if the square root of the AVE was greater than all the inter-construct correlation coefficients.

Finally, for evaluation of the concurrent validity of the ICQ-M, the Pearson’s correlation coefficient between the domains and total ICQ-M score and the total AAQ-II (M) score was computed, and a significantly higher correlation between the ICQ-M and the AAQ-II (M) score indicates good concurrent validity of the ICQ-M as the AAQ-II measures psychological inflexibility when facing negative life event, such as having cancer.

## Results

### Participants

Table 1 lists each participant's sociodemographic information, clinical characteristics, and mean total ICQ-M scores. More than half of the participants (*n* = 185, 53.3%) were middle-aged, between 46 and 65 years old, and 75% were females (*n* = 267, 76.9%). The low-income group (*n* = 269, 77.5%), which included individuals who made less than RM4500 per month, included around three-quarters of the participants. Clinically, over half of the individuals (*n* = 163, 47%) had breast cancer, and more than one-third (*n* = 140, 40.3%) were in stage II.

**Table 1:** Socio-demographic and clinical characteristics of the participants

Variables	Number of participants(n)	Percentage (%)
**Age:**		
18–25 years old	6	1.7
26–45 years old	97	28
46–65 years old	185	53.5
> 65 years	58	16.8
**Gender:**		
Male	79	22.8
Female	267	77.2
**Ethnicity:**		
Malays	279	80.6
Chinese	40	11.6
Indians	23	6.6
others	4	1.2
**Religion:**		
Islam	279	80.6
Buddhism	39	11.4
Hindu	23	6.6
Christian	5	1.4
**Monthly household income:**		
< RM 4,500	269	77.8
RM 4500-RM 11000	72	20.8
> RM 11000	5	1.4
**Marital status:**		
Married	283	81.8
Single/divorcee/widow/widower	63	18.2
**Education status:**		
Primary education or below	37	10.7
Up to secondary education	189	54.6
Tertiary education and above	120	34.7
**Time since diagnosis**		
3 months	61	17.6
3–6 month	65	18.8
6 months- 1 year	59	17.1
1–2 year	58	16.8
More than 2 years	103	29.8
**Types of cancer:**		
Breast cancer	163	47.1
Lung cancer	9	2.6
Head and neck cancer	84	24.3
Colon cancer	34	9.8
Others	56	16.2
**Stage of cancer:**		
Stage 1	51	14.7
Stage 2	140	40.5
Stage 3	116	33.5
Stage 4	39	11.3

The mean total ICQ-M was 54.96 (standard deviation (SD) = 9.14), while the skewness and kurtosis were -0.24 (SD = 0.13) and -0.20 (SD = 0.10), respectively. The mean acceptance and perceived benefit domain score was 37.78 (SD = 7.87), while the skewness and kurtosis were -0.39 (SD = 0.13) and -0.30 (SD = 0.10), respectively. Finally, the mean helplessness domain score was 17.19 (SD = 4.05), while the skewness and kurtosis were -0.33 (SD = 0.13) and -0.55 (SD = 0.17), respectively.

### Content validity index of ICQ-M

Table 2 provides an overview of the content validity index of the ICQ-M. All of the ICQ-M items' I-CVI values felt between 0.83 and 1.00. The ICQ-M had an S-CVI/Ave of 0.97. Last but not least, the ICQ-M's S-CVI/UA was 0.83.

**Table 2:** Content validity index (CVI) of the ICQ-M

Items	Expert 1	Expert 2	Expert 3	Expert 4	Expert 5	Expert 6	Expert in agreement	I-CVI	UA
Item 1	1	1	1	1	1	1	6	1	1
Item 2	1	1	1	1	1	1	6	1	1
Item 3	1	1	1	1	1	1	6	1	1
Item 4	1	1	1	1	1	1	6	1	1
Item 5	1	1	1	1	1	1	6	1	1
Item 6	1	1	1	1	1	1	6	1	1
Item 7	1	1	1	1	1	1	6	1	1
Item 8	1	1	1	1	1	1	6	1	1
Item 9	1	1	1	1	1	1	6	1	1
Item 10	1	1	1	1	1	1	6	1	1
Item 11	1	1	1	1	1	1	6	1	1
Item 12	1	1	1	1	1	1	6	1	1
Item 13	1	1	1	0	1	1	5	0.83	0
Item 14	1	1	1	0	1	1	5	0.83	0
Item 15	1	1	1	1	1	1	6	1	1
Item 16	1	1	1	1	1	1	6	1	1
Item 17	1	1	1	1	1	1	6	1	1
Item 18	1	1	1	0	1	1	5	0.83	0
Proportion relevance	1	1	1	0.22	1	1			
The average proportion of items judged as relevant across the six experts							S-CVI/Ave:	0.97	
							S-CVI/UA:		0.83

*I-CVI* Item-level content validity index, *UA* Universal agreement, *S-CVI/Ave* Average of the scale-level content validity index, *S-CVI/UA* Average of the scale-level content validity index across universal agreement among experts

### The face validity of the ICQ-M

When questioned about the semantic quality, comprehensibility of the words and phrases used and the instructions given, any redundancy of words used, and timing of the ICQ-M administration, 75% of the participants in the pilot study rated the criteria above as "appropriate." This was done while interviewing 20 native Malay-speaking cancer patients. A further 25% of respondents thought it was "very appropriate." There were no complaints regarding any deficiency in the above four factors assessed. Therefore, the expert group decided against making changes to the ICQ-M draft.

### Confirmatory factor analyses of the ICQ-M

In terms of the ICQ-M CFA assessment, a 2-factor model of the ICQ-M with merging of the acceptance and perceived benefit domains into a single domain, while the helplessness domain as another domain of the ICQ-M and all 18 items included, was not fitting ( ^2^/df = 3.007 with *p* < 0.001, CFI = 0.918, GFI = 0.896, TLI = 0.896, and RMSEA = 0.079). Then, a 3-factor model of the ICQ-M with item allocation similar to the original English language version of the ICQ was also not fitting ( ^2^/df = 3.723 with *p* < 0.001, CFI = 0.880, GFI = 0.863, TLI = 0.895, and RMSEA = 0.092). Finally, a 2-factor model of the ICQ-M with merging of the acceptance and perceived benefit domains into a single domain, while the helplessness domain as another domain of the ICQ-M and item 7 omitted was the best fitting model of the ICQ-M ( ^2^/df = 2.000 with *p* < 0.001, CFI = 0.958, GFI = 0.905, TLI = 0.955, and RMSEA = 0.059). Table 3 provides an overview of the CFA results of the ICQ-M.

**Table 3:** Confirmatory factor analysis of the Malay version of the Illness Cognitive Questionnaire (ICQ-M)

variables	**2-factor model of the ICQ-M**^**a**^	**2-factor model of the ICQ-M**^**b**^	3-factor model which follows the original ICQ
Chi-square/degree of freedom (χ2/df)	2.000*	3.007*	3.723*
Comparative fit index (CFI)	0.958	0.918	0.880
The goodness of fit index (GFI)	0.905	0.896	0.863
Tucker-Lewis Index (TLI)	0.955	0.896	0.895
Root mean square error of approximation (RMSEA)	0.059	0.079	0.092

^*^ = *p* < 0.001

^a^a 2-factor model with merging of the acceptance and perceived benefit domains into a single domain, with the helplessness domain as another domain of the ICQ-M and item 7 omitted

^b^a 2-factor model with merging of the acceptance and perceived benefit domains into a single domain, with the helplessness domain as another domain of the ICQ-M and all 18 items included

### The convergent and discriminant validity of the ICQ-M

Table 4 provides the results of assessing the convergent and discriminant validity of the ICQ-M, based on the 2-factor model of the ICQ-M that fitted the data the best. The AVE of the helplessness domain of the ICQ-M was 0.523, while the square root of the AVE was 0.723, which was greater than the interconstruct correlation coefficient between helplessness and acceptance and perceived benefit of 0.060. While for the acceptance and perceived benefit domain of the ICQ-M, the AVE was at 0.517, the square root of the AVE was at 0.719, which was larger than the interconstruct correlation coefficient between helplessness and acceptance and the perceived benefit of 0.060.

**Table 4:** Convergent and discriminant validity of the Malay version of the Illness Cognitive Questionnaire (ICQ-M) according to the best fitting 2-factor model of the ICQ-M in confirmatory factor analysis

Indicator variables	Latent variables	Standardized loading	Square of standardized loading	The sum of squared of standardized loading	Number of indicators	AVE	The square root of AVE	Inter-construct correlation
**Item 1**	HLP	0.680	0.462	2.614	5	0.523	0.723	HLP APB = 0.060
**Item 5**	HLP	0.672	0.452					
**Item 9**	HLP	0.663	0.440					
**Item 12**	HLP	0.908	0.824					
**Item 15**	HLP	0.660	0.436					
**Item 2**	APB	0.620	0.384	6.201	12	0.517	0.719	HLP APB = 0.060
**Item 3**	APB	0.614	0.377					
**Item 10**	APB	0.587	0.345					
**Item 13**	APB	0.789	0.623					
**Item 14**	APB	0.661	0.437					
**Item 17**	APB	0.693	0.480					
**Item 4**	APB	0.745	0.555					
**Item 6**	APB	0.780	0.608					
**Item 8**	APB	0.779	0.607					
**Item 11**	APB	0.784	0.615					
**Item 16**	APB	0.763	0.582					
**Item 18**	APB	0.767	0.588					

*HLP* Helplessness, *APB* Acceptance and perceived benefit, *AVE* Average variance extracted

### Concurrent validity of the ICQ-M

When the domains of the ICQ-M's Pearson's correlation coefficient and the overall AAQ-II score were evaluated, the helplessness domain of the ICQ-M was significantly moderate positively correlated with the total AAQ-II score (*r* = 0.435, *p* < 0.001). While the acceptance and perceived benefits domain of the ICQ-M was also significantly moderately positively correlated with the total AAQ-II score (*r* = 0.452, *p* < 0.001). Finally, the total ICQ-M score significantly positively correlated with the total AAQ-II score (*r* = 0.495, *p* < 0.001).

### Reliability of the ICQ-M

In the context of internal consistency of the ICQ-M, the Cronbach's alpha of the total score was 0.858, while that of the helplessness and acceptance and perceived benefits domains were 0.742 and 0.927, respectively.

## Discussion

Our study represents a noteworthy development in the realm of illness cognition research, with a specific focus on cancer patients in Malaysia. Through developing and validating the ICQ-M, we have introduced a valuable instrument for gaining insights into individuals' perceptions and thought processes concerning their illness. This innovative tool has profound implications for clinical practice and research, as it offers a culturally tailored means to assess illness cognition across a diverse ethnic population as in Malaysia. The ICQ-M has adequate content validity, as shown by the I-CVI of all its items being at least 0.83, the S-CVI/UA being higher than 0.8, and the S-CVI/Ave being higher than 0.9 [18–20, 26]. Twenty native Malay-speaking cancer patients participated in a pilot study to evaluate the ICQ-M's face validity. Of the respondents, 75% rated the semantic quality, comprehensibility, and administration time as "appropriate," and 25% rated it as "very appropriate," with no respondents criticising the use of redundant wording or sentences. This proved the ICQ-M's strong face validity.

In the context of its construct validity, the CFA performed confirmed the two-factor model of the ICQ-M, whereby the acceptance and perceived benefit domains were merged into a single domain, while the helplessness domain was maintained with item 7 omitted (Table 3). The differences in the language and cultures may explain the discrepancies between the factor structures of the original English and Malay version of the ICQ. Hence, the translated wordings in the ICQ-M may have a different meaning than the original English version. In the ICQ-M, item 7 was omitted as there were enormous similarities between the meaning of item 7 and item 15. When item 7 was omitted from the ICQ-M, the fitting of the 2-factor model was greatly improved (Table 3). When we inspected the best-fitting 2-factor model of the ICQ-M, the best-fit indicators were all acceptable (GFI, CFI, TFI, and RMSEA) except for the chi-square ( ^2^), in which the p-value was < 0.001. One of the limitations of chi-square statistics in CFA is that it is sensitive to sample size, whereby a large sample size will lead to the chi-square remaining statistically significant. The adequate sample size for CFA can be estimated as a cut-off of 200 subjects, or the sample size ratio to model variables should be at least 10:1 [27]. Our sample size in this validation study was 346 participants, and the ratio of sample size to model variables was 19:1. Hence, it is more appropriate to use the ratio of the chi-square to the degree of freedom ( ^2^/df) as a best-fit indicator rather than the chi-square itself. Moreover, it was suggested that a set of combined indices should be reported in assessing the best-fitting model in CFA, such as the chi-square, RMSEA, CFI, and SRMSR, rather than depending on chi-square statistics alone [27].

The ICQ-M had also achieved convergent validity as indicated by the AVE of the helplessness (0.523) and acceptance and perceived benefit (0.517) domains, which were greater than 0.5. In addition, the ICQ-M had also achieved discriminant validity as the square root of AVE for the helplessness (0.723) and acceptance and perceived benefit (0.719) domains were greater than their inter-construct correlation coefficient (0.06) (Table 4).

Regarding the concurrent validity of the ICQ-M, the AAQ-II was used as the comparator instrument. It was designed and validated to measure psychological inflexibility when facing a life event like cancer [23]. Our study reported that the domains and total score of the ICQ-M moderately correlated to the total AAQ-II score, indicating that the ICQ-M and its domains have similarities in their capability to measure acceptance of negative life event occurrence.

The internal consistency of the ICQ-M and its domains showed satisfactory to exceptional internal consistency (Cronbach's range from 0.742 to 0.927). It has been suggested that Cronbach’s α value between 0.70 to 0.95 is acceptable [28]. Regarding internal consistency, the ICQ-M domains and the original English ICQ were comparable (the latter's Cronbach's α ranges from 0.84 to 0.91) [21]. Similarly, the internal consistency of the ICQ-M was also comparable to that of the Korean version of the ICQ (Cronbach’s α range from 0.79 to 0.86), and that of the ICQ adapted for use to assess illness perception and cognition in parents of children with cancer [8, 29].

There were a few limitations of this validation research. First, the research sample's gender distribution and cancer types were not indicative of Malaysia's overall cancer population. Hence, this has an impact on how generalizable the research results are. Similarly, the cancer patient recruitment only involved a single center which may again affect the applicability and generalizability of the study findings to be representative of the Malaysian cancer population.

Despite its limitations, this study effectively translated, adapted, and validated the ICQ-M to manage cancer patients in Malaysia. Now, patients with cancer may use the ICQ-M to gauge the illness perception of cancer patients, which is an essential determinant of the mental state of the patient and their compliance with cancer treatment, quality of life, and prognosis. Psychotherapy or other effective psychosocial interventions can be administered to cancer patients if their poor illness acceptance and a high degree of helplessness affect their mental state and disrupt their compliance with cancer treatment to improve the outcome of their cancer management. Moreover, the translated and validated ICQ-M can also be adapted and validated in future studies to be applied for measuring illness perception of other chronic illnesses in the Malaysian population.

## Conclusion

The original English version of the ICQ has been successfully translated to Malay, and the ICQ-M exhibited good reliability, such as internal consistency, and decent validity, such as the face, content, convergent, discriminant, construct, and concurrent validity. The CFA of the ICQ-M confirmed that the ICQ-M consists of 17 items designated to two domains. The ICQ-M is now available to measure the illness perception and cognition among cancer patients in Malaysia.

## Relevance for clinical practice

ICQ-M holds significant relevance for the clinical practice among cancer patients in Malaysia. The ICQ-M helps healthcare workers to get insight into patients' mental states and attitudes toward cancer by offering a trustworthy and valid instrument to evaluate disease perception and cognition. These results can inform personalized treatment plans, help to understand patients, and guide the implementation of psychotherapeutic interventions to improve illness acceptance and reduce helplessness. Ultimately, the ICQ-M improves patient outcomes and overall cancer management in clinical settings.

## Data Availability

The datasets used and/or analysed during the current study are available from the corresponding authors on reasonable request.

## Abbreviations

ICQ-M: Malay version of the Illness Cognition Questionnaire
B-IPQ: Brief Illness Perception Questionnaire
SRM: Self-regulatory model
AMDI: Advanced Medical and Dental Institute
EFA: Exploratory factor analysis
I-CVI: Item-level content validity index
UA: Universal agreement
S-CVI: Scale-level content validity index
AAQ-II: Acceptance and action questionnaire version 2
CFA: Confirmatory factor analysis
AVE: Average variance extracted
RM: Ringgit Malaysia
SPSS: Statistical Package for Social Sciences
2/df: Chi square to degree of freedom ratio
TLI: Tucker-Lewis index
CFI: Comparative fit index
GFI: Goodness of fit index
RMSEA: Root mean square error of approximation
